# General practitioners’ perspectives on relocating care: a Dutch interview study

**DOI:** 10.1186/s12875-024-02425-1

**Published:** 2024-05-25

**Authors:** L.J. Damen, L.H.D. Van Tuyl, B. J. Knottnerus, J.D. De Jong

**Affiliations:** 1 Nivel, The Netherlands Institute for Health Services Research, Utrecht, The Netherlands; 2 CAPHRI, Maastricht University, PO Box 616, 6200 MD Maastricht, The Netherlands

**Keywords:** Primary care, General practitioners, Relocating care, Healthcare system, Qualitative research

## Abstract

**Background:**

Healthcare systems around the world are facing significant challenges because higher costs and an increase in demand for care has not been matched by a corresponding growth in the health workforce. Without reform, healthcare systems are unsustainable. Relocating care, such as from hospitals to general practices, is expected to make a key contribution to ensuring healthcare remains sustainable. Relocating care has a significant impact upon general practitioners (GPs). Therefore, we investigated which care, according to GPs, could be relocated and under which conditions.

**Method:**

GPs were recruited through Nivel’s GPs network on eHealth and innovation, located in the Netherlands. One exploratory focus group and 12 in-depth interviews were conducted. Interview transcripts were analyzed using the qualitative research principles of thematic analysis.

**Results:**

According to the participants, some diagnostic and follow-up care could be relocated from hospitals to GPs once certain prerequisites are fulfilled. An important condition of relocating care from the hospital to the GP is that GPs have sufficient time to take over these tasks. The types of care that can be relocated from the GP to other settings are those questions where the medical knowledge of the GP can offer nothing extra or where problems in navigating the health system cause patients to either turn to, or stay with, their GP.

**Conclusion:**

Care should first be relocated from the GP to other settings before attempting to organize the relocation of care from the hospital to the GP. When this, and other conditions are met, some diagnostic and follow-up care can be relocated from the hospital to the GP.

**Supplementary Information:**

The online version contains supplementary material available at 10.1186/s12875-024-02425-1.

## Introduction

The accessibility and financial stability of healthcare are being put at risk in many parts of the world due to increasing demand for care, rising healthcare expenditures, and shortages in the workforce [1–6]. Therefore, reforms of healthcare systems aimed at improving healthcare efficiency are necessary to ensure the future sustainability of healthcare [7]. Estimates suggest that one fifth of health spending could be channelled towards better use [7]. This can be achieved through various means. The number of patients who receive low-value or unnecessary care could be reduced. The same care could also be provided using fewer resources, for instance by providing care in more cost-effective settings rather than in hospitals. Finally, administrative processes that add no value could be reduced. This article focuses on providing care with fewer resources by relocating it to more cost-effective settings. Hospital care, in general, counts for the largest part of healthcare costs in OECD (Organisation for Economic Co-operation and Development) countries [8]. For certain procedures that do not need the staff or environment of the hospital, general practitioners (GPs) can generally provide care at less expense than hospitals [7, 9–12]. These procedures may include minor surgery, for example, placing an intra-uterine device, the removal of stitches, or performing gastric ultrasounds. Therefore, it is anticipated that reducing hospital-based care and relocating this care closer to people’s homes could contribute significantly to the sustainability of healthcare [7]. We investigated which care, according to GPs, should be relocated to them from the hospital given the significant impact such a change could have upon primary care.

It was anticipated that GPs would first need to relocate some of their existing responsibilities to alternative settings or individuals in order to make relocating care from the hospital to the GP possible. This would allow them to free up sufficient time to assume their additional responsibilities. This expectation was drawn from existing literature, which highlights the significant pressure on GP care in numerous countries with approximately four out of five GPs in the UK, Germany, and the Netherlands expressing little to no satisfaction with their current workloads [13]. In the current study we therefore also investigated which care can be relocated from the GP to alternative settings. These alternative settings may encompass eHealth, other healthcare professionals or citizens, by improving self-care. When care is relocated from the GP to another healthcare professional (HCP), to self-care, or to eHealth, it also entails a change in the care delivery environment. This is why we refer to it in this article as relocating care to alternative settings.

In order to ensure the success of relocating care, it is crucial to investigate how GPs think about relocating care. GPs serve as experts in their field, possessing insights into which care can be feasibly relocated while taking into consideration their own skills and capabilities [14]. Existing studies on this subject often lack the inclusion of the GPs’ perspective and instead emphasize cost considerations [15–17]. Some studies focus on the citizen’s perspective [18, 19] or other stakeholders like hospital specialists [20, 21]. Alternatively, certain studies concentrate solely on specific services like one-and-a-half-line care [22–24] or particular diagnoses, such as incontinence and cancer [19–21, 25–27]. From the few studies into the GPs perspective on relocating follow-up cancer care, barriers identified by the GPs included concerns about their skills and communication with the oncology specialists [19, 20, 25, 27].

In order to enhance our comprehension of GPs’ attitudes and requirements concerning relocating care, our objective was to acquire in-depth information on GPs’ perspectives on this matter. This study addresses the following research questions: (1) According to GPs which care can be relocated? (2) According to GPs, what are the conditions under which care can be relocated?

## Method

### Setting

This study took place in the Netherlands, where GPs have a central role in the healthcare system. They are the main point of entry to the rest of the healthcare system and thus perform a gatekeepers function [28]. GPs take on a medical advocacy role for individual patients. They monitor the health of the patients and they coordinate patient care. They are the first point of contact when citizens have a medical issue.

### Design

We used a qualitative design, including one exploratory focus group discussion with six GPs, followed by 12 semi-structured in-depth interviews.

### Sample and recruitment

The GPs were recruited using convenience sampling through the GPs network on eHealth and innovation of the Netherlands Institute for Health Services Research (Nivel). This includes approximately 120 GPs who have previously demonstrated an interest in participating or providing insights on research topics related to eHealth, innovation, and sustainability. An email invitation was sent to this network allowing GPs to express their interest in participating in this specific study. The date for the exploratory focus group was selected based on the availability of the majority of GPs, and it took place in December 2021, with six GPs participating.

For the subsequent interviews in March 2023, the six GPs who participated in the exploratory focus group were excluded. From the remaining pool of respondents to the email invitation, a total of 21 GPs expressed interest. We initially scheduled 12 interviews based on the literature suggesting that this number is typically adequate to achieve saturation [29]. The remaining nine GPs who had expressed interest were kept in reserve and could be approached if saturation was not achieved after 12 interviews. After conducting the initial 12 interviews, saturation was indeed achieved. Therefore, the remaining nine GPs were not approached for interviews.

### Data collection

The exploratory focus group was conducted online and spanned 90 minutes. A semi-structured format was followed, (Appendix A).

The interviews also followed a semi-structured format (Appendix B). The design of the interview guide drew upon insights gained from the exploratory focus group. Moreover, the guide was compiled by all authors using relevant literature [10] and benefiting from the diverse backgrounds of each author. Specifically, one author actively practices as a GP, another possesses expertise in healthcare system and policy, a third author brings over fifteen years of experience in qualitative research as a methodologist, while the fourth author has a background in sociology and prior experience as a paramedic. There were two pilot tests for the interview guide with two different GPs. Their feedback was incorporated into the guide.

We refined the interview guide’s questions during the process of data collection, in order to ensure that we were given the in-depth information needed to answer the research questions. We noticed after eight interviews that we had less information about relocating care from the hospital to the GP than about relocating care from the GP to other settings. We, therefore, added additional in-depth questions to the interview guide regarding relocating care from the hospital to the GP. The interviews were conducted by the first author online and had a maximum duration of 45 min. The sessions were recorded on audio and accompanied by field note entries immediately following the interview.

### Analysis

A verbatim transcription of the exploratory focus group was produced, and a report was generated. This report was shared with the participating GPs for member-checking, allowing them to provide feedback. The exploratory focus group was performed to inform the structure of the interviews, therefore, it was decided not to delve extensively into the results of the exploratory focus group discussion in the results section.

The interviews were transcribed verbatim and anonymized by the first author (L.D.). Subsequently, the data collected was imported in the software program MaxQDA 2022 for analysis. The transcripts of the interviews were subjected to thematic analysis, involving the following steps: becoming familiar with the data; generating initial codes; searching for themes; reviewing themes; defining and naming themes; and, producing the report [30]. Thematic analyses were used because this is a flexible and systematic method for identifying themes within qualitative data. The first four transcripts were analysed independently by both the first and second authors (L.D. and L.T.). The initial codes derived from these independent analyses were subsequently discussed between the two authors until a consensus was reached. A concise codebook was developed based on those initial codes (Appendix C). This codebook was discussed within the research team and refined after consultation. Next, all further transcripts were analysed by the first author (L.D.). Whenever uncertainty arose, or before introducing a new code, L.D. engaged in discussions with L.T. A new code was added once a mutual agreement had been reached. After coding the transcripts subsequent to consultation with the research team, the definitive themes, as presented in the results section, were established. The quotes that are shown in the results section are translated from Dutch.

### Trustworthiness

The four key criteria for assessing trustworthiness are: credibility, generalizability, dependability and confirmability [31]. Below, we will elaborate on additional measures we undertook to assess trustworthiness, beyond those outlined in the method section. To enhance credibility, we consistently compared new data with previously collected information to identify similarities and differences during the data coding process. Additionally, there was a thorough discussion within the research team about all the interim and final codes, themes, and results. We thus employed triangulation to ensure their credibility. Co-author B.K., who is a practicing GP, verified the completeness and accuracy of the findings. He helped interpreting the results and facilitating discussions on the subsequent steps, thereby bolstering the credibility of our study. Moreover, the interviews confirmed content from the exploratory focus group discussion, further enhancing credibility. In addition, we carried out ‘peer debriefing’ with a group of peer researchers who were not involved in the study.

We have enhanced how generally applicable the results were by providing detailed information about the participant characteristics, and the specific setting in which these GPs work. These descriptions facilitate the assessment of how transferable our results are to other contexts.

To enhance how dependable our study is, we followed both the ‘criteria for reporting qualitative research (QOREQ)’ [32], and the guidelines for thematic analyses [30]. We also ensured a detailed reporting process.

Lastly, we fortified confirmability by including verbatim statements from participants in the results section.

### Ethical procedures

Approval by a medical ethics committee is not needed for non-experimental exploratory focus group or interview data involving experts, in this case, GPs, according to Dutch law [33]. Data collection has been performed in accordance with the Declaration of Helsinki. The anonymity of the GPs was strictly preserved through the process of data entry and analysis.

Participating GPs received an informed consent form a few days before the interview. Before recording the interview we asked if the GPs had any questions with regard to the informed consent. Subsequently, they were asked to confirm their agreement with the terms of the informed consent. This confirmation was obtained on the recording. All participants gave verbal informed consent.

## Results

We conducted an exploratory focus group with six GPs, followed by 12 in-depth interviews with GPs. Their background characteristics are presented in Table 1.

**Table 1:** Background characteristics of GPs in the exploratory focus group (*N* = 6) and interviews (*N* = 12)

	Exploratory focus group	Interviews
Gender		
• Male	4	9
• Female	2	3
Age		
• 40–49	2	4
• 50–59	1	3
• 60 and older	3	5
Type of general practice the GP is working in		
• Solo practice	1	4
• Duo practice		5
• Group practice	1	1
• Practice within a health centre	4	2
Years of experience working as GP		
• 6–10		1
• 11–20		5
• More than 20		6
How urban is the area in which the practice is located*		
• Highly urbanized	1	7
• Slightly urbanized	4	2
• Less urban/rural	1	3

*The level of urbanization is determined by the average ambient address density, categorized as follows: (1) Highly urbanized area: with an average ambient address density of 1500 or more addresses per square kilometer. (2) Moderately urbanized area: characterized by an average ambient address density ranging from 1000 to 1500 addresses per square kilometer. (3) Less urban/rural area: exhibiting an average ambient address density of 999 or fewer addresses per square kilometer [34].

### GPs feel the need to tackle existing burdens before taking on new responsibilities

In the exploratory focus group, GPs highlighted the need for relocation of care away from the general practice, rather than exploring options for relocating care from hospitals to GPs. Participants stressed the importance of addressing existing burdens before considering additional responsibilities:

‘But it is mostly about cleaning up the shed before putting new items in.’

– Participant 1 exploratory focus group (male, aged 60 years or older).

This insight guided the structure of subsequent interviews, with initial inquiries focused on identifying tasks that could be delegated from GPs to create space for discussing potential new responsibilities. The areas of care identified for potential relocation by the exploratory focus group participants align with those mentioned by GPs during the interviews. The areas identified through the interviews are described in detail below.

### Necessity for organizational changes

Almost all participants in the interviews recognized the necessity for organizational changes to ensure the general practice care was sustainable. This motivated their participation in this interview. While not all GPs saw relocating care as the ultimate solution to keep healthcare sustainable, a majority identified potential advantages in relocating care. They believed that the quality of care could be enhanced by relocating care from the GP to other HCPs who were specifically trained, such as social workers. In addition, by relocating care from the hospital to the general practice, which is often closer to people’s homes, patients are treated with a more holistic approach compared to care provided at the hospital. Furthermore, a subset of GPs believed that their pleasure in their work would increase if they could do more minor interventions. However, while recognizing the benefits of relocating care from the hospital to general practice, most GPs emphasized the need to relocate care away from the general practice, as was also mentioned during the exploratory focus group. They pointed that without relocating this care, they would lack the necessary time to accommodate new tasks from the hospital.

**Fig. 1:**
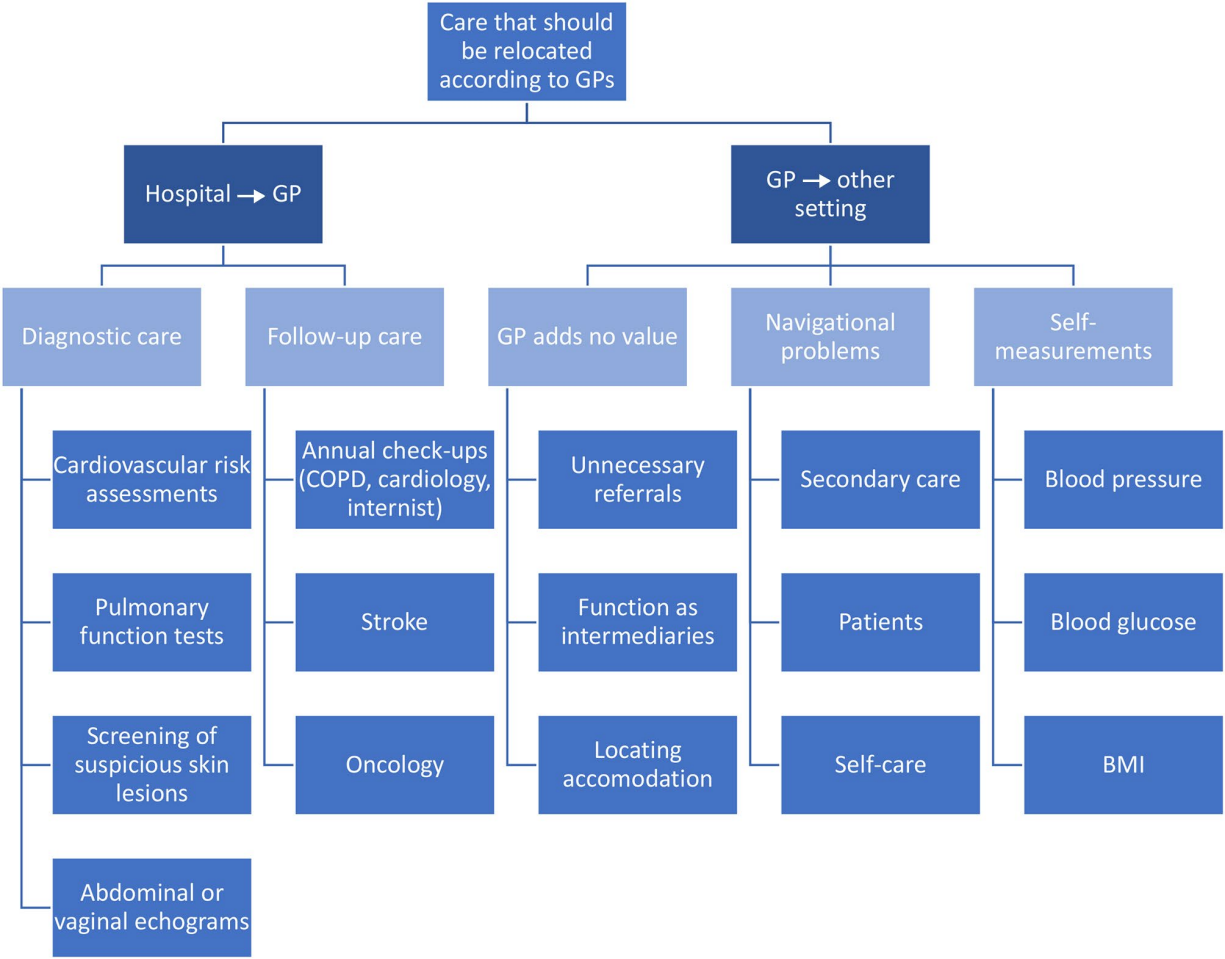
Care that could be relocated according to GPs in the interviews

The interviews identified themes within two domains: (1) types of care that could be relocated from the hospital to the GP; (2) types of care that could be relocated from the GP to other locations. The themes within these domains are discussed in the next sections and summarized in Table 2. Moreover, Fig. 1 illustrates the care that could potentially be relocated, as per the insights gathered from the interviews with general practitioners.

**Table 2:** Themes identified in the interviews

Types of care to be relocated from the hospital to the general practice	Types of care to be relocated from the general practice to other settings
1) Diagnostic care	1) GP adds no value
2) Follow-up care	2) Navigational problems
	3) Self-measurements

## Types of care to be relocated from the hospital to the general practice

The participants highlighted that a substantial amount of healthcare has already been relocated from hospital to GP. This includes integrated care for diabetes and chronic obstructive pulmonary disease (COPD). In combination with the fact that most participants emphasized that they currently lack the necessary time to assume more tasks from hospitals, the participants found it difficult to name concrete examples of care that could possibly be relocated in the future. Despite this fact there was some care that could be relocated according to some of the participants, which will be discussed in the following sections.

### Diagnostic care

Initially, a few of the GPs participating saw some possibilities for relocating diagnostic care from the hospital to the GP. They mentioned several examples of diagnostic procedures that could be feasibly managed by GPs. These included cardiovascular risk assessments, pulmonary function tests, screening of suspicious skin lesions, and abdominal or vaginal echograms. To facilitate this, GPs would require access to the necessary instruments to conduct these diagnostic tests. Additionally, the further development of certain instruments would be essential, such as a screening tool for skin lesions that highlights areas that require attention from the GP:

Quote 1: ‘For example, the abdomen or stomach or…. I think vaginal ultrasounds are suitable too. But that is also because those devices are getting better, the device can indicate that everything is in order and can tell you where to look or feel a bit more.’

– Participant 2 (female, aged 50–59).

### Follow-up care

The second form of care that could be relocated, according to some participants, is follow-up care. They named annual check-ups for COPD, cardiology and with an internist as care that could be relocated to the GP. Furthermore, they said that oncology patients and patients who have had a stroke can be relocated to the GP at an earlier stage. Patients who had a stroke are supervised by the hospital for a year after they have had a stroke. Subsequently, they will be supervised by the GP, who offers exactly the same guidance as the hospital did. One of the GPs said that patients could also be relocated to the GP after three months, provided their condition is stable.

However, some of the participants said that fostering referrals to the GP for this kind of care first requires a change in the financing system. They said that hospitals may be reluctant to relinquish such patients, as these check-up consultations take little time for them and generate revenue for the hospital:

Quote 2: ‘There are still many patients in secondary care who do not belong there. These patients are convenient for generating revenue, as they consume minimal consultation time. They are considered easy patients. They keep them there to ease the workload, while maintaining revenue. (…) Once people have atrial fibrillation, but they are stable, then, according to the guidelines, they are allowed to go back to the GP. I had this checked and out of 100 patients, 20 could go back to the GP. Because their condition is well-controlled, no additional hospital care is needed.’

– Participant 5 (male, aged 60 or older).

The prerequisites can be applied both to relocating diagnostic care and follow-up care outlined by the GPs. Therefore, the prerequisites will be discussed together in this section.

First of all, practical considerations emerged as important prerequisites. Including, the most frequently mentioned condition, that GPs need to have sufficient time to take over tasks from the hospital. This is required in order to alleviate first their workload and thus enable them to accommodate additional responsibilities. Furthermore, GPs noted the need for adequate physical space, financial resources, and a sufficient workforce, in order to accommodate the tasks associated with relocated care. Secondly, the participants mentioned that there needs to be good collaboration between the GP and the specialist. There needs to be a seamless transfer of the care with clear and rapid communication channels deemed essential. GPs want to have advice from the specialist when needed, along with the ability to refer patients back to the hospital promptly when necessary, without being constrained by waiting lists. Thirdly, participants highlighted the significance of stable government policies (quote 3). Concerns were expressed that while GPs may initially receive subsidies for taking on tasks from hospitals, these incentives could, potentially, be withdrawn after a few years.

Quote 3: ‘There needs to be a structural adjustment in financing to prevent situations where, after three years, the budget is depleted. They then assume that care is relocated, so it stays with the GP, but we do not have the necessary budget to support it anymore.’

– Participant 7 (male, aged 50–59).

Lastly, most of the GPs expressed strong enthusiasm for teleconsultations with specialists. They believed that making use of these could lead to a reduction in referrals to hospitals. Moreover, they emphasized that these virtual collaborations enhance their own knowledge base through interactions with the specialist. Some GPs also thought that one-and-a-half-line care would augment knowledge as it involves a close collaboration between general practitioners and medical specialists outside the hospital. Thus, specialists provide treatment advice to GPs, with the GP maintaining control over the patient’s care. However, others were concerned it might actually diminish knowledge due to the lowered threshold for brief specialist consultations, potentially resulting in more specialist consultations than before. Moreover, several GPs thought that the patient volume within their practice might not be sufficient to justify having a specialist within the practice structure. Additionally, several GPs expressed scepticism about the added value of one-and-a-half-line care in comparison to directly referring patients to secondary care, where hospitals can then refer them back as necessary.

## Types of care to be relocated from the general practice to other settings

Participants highlighted the importance of relocating care from GPs to other settings as a significant prerequisite for reducing their workload and making it possible to relocate care from the hospital to GPs. The participants identified several aspects of care that they believed should be relocated, these will be discussed in the next sections.

### GP adds no value

Participants expressed a wish to relocate care where their knowledge and expertise do not provide any added value to the care they provide. They refer to tasks that consume a considerable amount of their time and sometimes are unnecessary or could be undertaken by others. Specifically, many participants mentioned that this included writing unnecessary referrals or notes, requested by another HCP or by patients themselves, even though the GP has not been actively involved in the patients’ treatment nor even the patient (quote 4). Some participants indicated that this is occasionally driven by insurance requirements, some of which they believe should be abolished (quote 5), or sometimes as a preference from other HCPs who wish to share responsibility.

Quote 4: ‘Then I thought that is actually crazy, because you guys [referring to a coaching team] make a treatment plan, you know the details of the child. I do not. I had not seen the child for two years. And in the end I have to make a referral. It would certainly unburden the GP if we do not have to write all kinds of crazy notes for things that do not make sense at all. Because I am not in charge of that care. I am not in charge of anything at all, except signing.’

– Participant 10 (male, aged 40–49).

Quote 5: ‘Some insurers ask for signatures that make no sense, for things that are unnecessary. For example, we have to write a yearly note for an artificial leg. If a leg is amputated it does not come back anymore, so why should we declare this every year?’

– Participant 8 (female, aged 40–49).

Some of the participants suggested a potential solution could involve enhancing direct accessibility to a wider range of HCPs:

Quote 6: ‘There are several situations where people request referrals from me, because they intend to seek care from other professionals, such as podiatrists, pedicurists, or skin therapists… And that should actually be allowed to be directly accessible.’

– Participant 2 (female, aged 50–59).

Moreover, those HCPs who are already directly accessible should shoulder responsibility by making use of this direct access and refraining from requesting referrals.

Furthermore, many participants noted that they receive a significant number of referrals from secondary care, where the GP does not contribute value with their knowledge or expertise. For instance, GPs are often requested to conduct annual blood tests on patients already undergoing treatment at the hospital. When the test results are normal, the GP’s involvement is minimal. However, if the results are abnormal, then the patient needs to be referred back to the hospital. Some participants expressed the sentiment that they merely function as intermediaries in this process, without adding meaningful medical input. A few participants mentioned that they would not mind performing these tasks if they were also able to provide follow-up care, thereby extending their role beyond that of a mere messenger:

Quote 7: ‘I am opposed to the idea of patients receiving care and coaching from the hospital and, meanwhile, I am only responsible for assessing oxygen levels. It is a bit of an all-or-nothing principle. We are not the assistant of the hospital specialist.’

– Participant 10 (male, aged 40–49).

Participants also highlighted the time-consuming nature of locating accommodation for patients in mental health or home care, a task they deemed unnecessary and unrelated to a GP’s core responsibilities. Home care organizations and mental health services are often overwhelmed, leading to lengthy waiting lists, which in turn make it challenging for GPs to make patient referrals. Consequently, GPs spend substantial time searching for suitable placements for their patients. GPs emphasized two key points regarding the burden of finding accommodation. Firstly, they stressed that this responsibility should not be placed solely on GPs, but should also involve home care and mental health organizations. Secondly, they pointed out that sourcing appropriate accommodation could be effectively delegated to other individuals, as it may not always necessitate the specialized expertise of a GP.

### Navigational problems

GPs observe that many inquiries are directed to them, or remain their responsibility, due to issues related to navigation. These navigational problems manifest at various levels. Firstly, there are problems within secondary care, for example it is often unclear which is the appropriate ward or department for a patient (quote 8 and 9). GPs mentioned that care sometimes is too specialized, particularly for patients with multiple medical issues that do not clearly fit into a single specialism. Consequently, there is a reluctance to assume responsibility for such cases, leaving them the responsibility of GPs as these are the first tier of the healthcare system. GPs mentioned that other HCPs should take responsibility, or that responsibility for these kinds of patients should be shared between the GP and the specialist.

Quote 8: ‘The crisis team said this woman needs to be somatically assessed and then hospitalized. The internist said the patient belongs in a psychiatric ward. However the psychiatrist, wants us to go to the crisis team. This is an example of how the system operates, placing responsibility on the GP. I had to spend an hour on the calls during consultation hours.’

– Participant 6 (male, aged 60 or older).

Quote 9: ‘We referred someone for suspected PTSD (post-traumatic stress disorder) and the response we received was that this patient does not fully comply and they just sent the patient back. But nobody doubts the fact that there is something wrong with that person. I really do not care whether you call that PTSD or something else. I would prefer them to say that they will examine the possibility of PTSD based on my concerns and if it does not match PTSD additional diagnostics will be performed’

– Participant 4 (male, aged 40–49).

Secondly, people have navigational problems that is not knowing where they should go. This is because they often lack clarity about which HCP to contact and so they end up asking their GP for guidance. Also, not all citizens are aware of which HCPs are directly accessible, resulting in visits to their GP to request referrals. This commonly arises with questions related to the physiotherapist or social worker. As a potential solution, some GPs suggested the implementation of a centralized information point within the local authority. This central point would serve the purpose of guiding individuals to the appropriate healthcare resource:

Quote 10: ‘I think there is a role for the government, in particular to provide good information about the resources available. And then it would be best to think about creating a central point where people can go with a question. And then the people there can say, this is for youth care, for this you have to go to the GP, this you have to arrange with social services, and that you have to arrange with the housing department.’

– Participant 3 (male, aged 60 or older).

Thirdly, inquiries that end up with the GP due to navigational problems are related to self-care. Self-care could be applied to a lot of health complaints that come to the GP, for example when citizens have a cold (quote 11). However, according to the GPs, many citizens do not know what they can do themselves, or are hesitant to assume responsibility for their own health. GPs mentioned that this is also due to changes within society, whereby citizens overwhelm their GP and feel minimal restraint in seeking their services. They add to this that the general practice has also become very approachable and that GPs find it difficult to say ‘no’ these days. A subset of participants expressed the view that GPs have a role in clarifying which questions should be addressed by them and which should not. Others believe that this responsibility should lie with the government or health insurers. Also, it was mentioned that the government could do more with regard to health promotion by supporting initiatives such as creating playgrounds, backing sports clubs, and imposing taxes on unhealthy foods. These efforts would mitigate health problems and reduce the number of patients seeking GP consultations. In addition, most participants thought that citizens should assume greater responsibility for their personal health. For instance, they could follow advice provided on GPinfo (‘*thuisarts.nl*’), an independent website for health information developed and maintained by the Dutch college of GPs [35]. Nevertheless, it was also said that this might pose challenges for certain individuals, depending on factors such as their level of health literacy.

Quote 11: ‘For example, when it comes to children and colds, when should you reach out to the GP? Frequently, the assistant’s response is that individuals can locate the information themselves by simply checking the website.’

– Participant 1 (male, aged 60 or older).

GPs are aware of these navigational problems and questions that come to them where the they can add no value to care. However, many of them struggle with a contradiction when thinking of relocating care. GPs see themselves as linchpins. They are aware of all the patient information available, they have many contacts with HCPs and patients’ networks, and they have extensive medical knowledge. They like to keep an overview and control the direction of patient care. While they name several things that should be relocated from the GP to other places, they, at the same time, find it challenging to relinquish these tasks entirely:

Quote 12: ‘Doctor I have mice in my house… Or my cat has diarrhoea. Then I explain that I am not the right person for these concerns. I would not think of calling my GP if I have mice. It is really bizarre what kind of questions we receive.’

(…)

But I think we are a linchpin, given our comprehensive knowledge. And if we can refer people to the right person, then it is fine, but now the problem stays with us. They continue to call and say that those mice are still there.’

– Participant 9 (female, aged 40–49).

### Self-measurements

Some participants identified the significant potential of the concept of patient self-measurement as a promising strategy to lighten the workload of GPs. Self-measurements could be applied for, for instance, blood pressure or blood glucose. Participants mentioned that self-measurements are still in the development phase, but thought that implementing more self-measurements could save the GP a lot of time. Another advantage that comes with this is that measurements are more exact, because patients can measure values multiple times (quote 13). Also the GPs claimed it allows patients to take more control of their own disease. In addition, self-measurements could play a role in prevention. When patients enter their BMI (Body Mass Index) into the system, the GP can then respond to this during a consultation (quote 14).

Quote 13: ‘Firstly, it gives more detailed and accurate information and, secondly, blood pressure at home is always a bit lower anyway, which leads to a reduction in the medication we need to prescribe.’

– Participant 12 (male, aged 60 or older).

Quote 14: ‘There are a lot of people who are overweight coming to our consultation for colds, or knee pain, or whatever and then you do not have a conversation about being overweight. But if they enter weight information via a patient portal, we would have access to this data and can start screening.’

– Participant 10 (male, aged 40–49).

## Discussion

The research questions within this study were: (1) According to GPs which care can be relocated? (2) According to GPs, what are the conditions under which care can be relocated? To address these questions, an exploratory focus group involving six GPs was conducted in December 2021. It became evident during this discussion that, before discussing the relocation of care from hospitals to GPs, it was crucial to determine what care could potentially be relocated from GPs to other healthcare settings. Insights gathered from this discussion played a pivotal role in shaping the interview guide for subsequent interviews.

Subsequently, twelve interviews were conducted in March 2023 with practicing Dutch GPs, aimed at exploring their perspectives on care relocation. While not all GPs believed that relocating care is the sole solution for sustaining the healthcare system, the majority recognized the advantages of such relocation. They believed it would enhance the quality of care, increase their professional satisfaction, and improve patient outcomes.

Care that could be relocated is, according to the GPs, follow-up care, diagnostic care, care where the GP adds no value, care that ends up at the GP due to navigational problems, and care that could be replaced by self-measurements. Various conditions were identified for relocating care. For relocating care from the hospital to the GP it was emphasized that GPs must have sufficient time. Also other practical conditions like adequate physical space were mentioned. Moreover, good communication and collaboration between GPs and specialists were highlighted as essential. To relocate care where the GP adds minimal value, a condition is the abolition of certain unnecessary referrals requested by insurance companies, and other healthcare professionals taking responsibility for their tasks within the system. To relocate care that ends up at the GP due to navigational issues, it is essential to establish accessible resources that can guide individuals in the right direction. Additionally, educating citizens about which questions are appropriate for GPs and which are not was deemed important.

### Comparison with previous literature

Follow-up care for cancer was often mentioned by the participants as care that could be relocated, a sentiment echoed in existing literature [19–21, 25, 27]. Literature has shown that clinical outcomes for follow-up cancer care are similar between general practice and hospital care [36–38]. Effective communication between GPs and specialists emerges as a crucial factor for this transition, as emphasized by both the participating GPs and previous research across various countries [19, 20, 27]. However, current transfers from hospitals to GPs do not always occur as efficiently as anticipated in several high-income countries [8]. This suggests healthcare systems are failing to deliver effective care. GPs in the Netherlands, Germany, Sweden, and the United States score relatively low compared to seven other high income countries with regard to receiving information from specialists, for example about changes made to their patients medication or care plans [39]. Therefore, an important condition for organizing the relocation of care is to improve the communication and collaboration between specialists and GPs.

GPs participating in our study did not express concerns about lacking the necessary skills when care is relocated from hospitals to their practices. This fear of inadequate skills is cited as a significant barrier in healthcare literature [25, 27]. However, our study revealed that the GPs felt confident in their abilities to assume care responsibilities from hospitals. They mentioned either possessing the requisite skills already or being open to undergoing additional training to acquire them. Consequently, they did not harbor apprehensions regarding their capability to manage relocated care effectively. Further investigation into this matter is warranted.

Most GPs found their current workload too high and therefore did not think care could be relocated from the hospital to the GP. This observation aligns with previous literature underscoring GPs’ dissatisfaction with their workload, a trend evident across various nations, including the UK, Germany, Sweden, and the Netherlands [13, 40]. This could explain why we gathered more information about relocating care from the GP to other settings during the interviews rather than from the hospital to the GP. This underscores the necessity of initially organizing the relocation of care from the GP to other settings in order to organize relocating care from the hospital to the GP.

The participants believed that the care that can be relocated from the GP to other settings is care in which the GP adds no value, medical or otherwise, or is care that is coming to the GP due to navigational problems. These results support the outcomes of the redefined core values of the Dutch GPs [41]. The old core values contained the core task ‘generalism’, this was changed into ‘medical generalism’. GPs made clear that they are not responsible for solving social and lifestyle-related problems, and that they want to add medical knowledge.

One of the other core values is the ‘coordination of care’, which includes helping patients to find their way within healthcare and social services [41]. Navigational problems in this study also included patients who could not find their way. GPs interviewed said it would save them time if patients went directly to the right place. However, at the same time, they think of themselves as linchpins who should coordinate care for patients. This seems contradictory. However, when seen through the lens of their core objective, of adding medical value, it seems logical that the coordination of care should be relocated. By pointing the way to another professional, the GP adds no medical value. This task could effectively be handled by someone else, for example by a centralized information point within local authorities. When pressure at the general practice is high, this could be relocated. In addition, some GPs do not seem to have problems with directing patients to the right place, but they have problems with the fact that the question is not always followed up by other HCPs or citizens after referral and so the question remains with the GP.

This study has offered valuable insights into the perspectives of GPs regarding the relocation of care. It is important to involve GPs in policy making in order to close the gap which often exists between research, practice, and policy [14]. Engaging healthcare providers in policymaking can enhance the practical application of evidence, thereby fostering more effective policies. This was also stated by some participants. They mentioned the disconnect between those who create policy and those who implement it. Furthermore, participation in decision-making improves the feeling of equality for different parties [14]. This inclusive approach can lead to increased satisfaction and collaboration. This study, thus makes an important contribution to the effective organization of relocating care.

To date, limited research has been conducted into the perspective of GPs on the relocation of care. Previous research exclusively focused on specific diagnoses or forms of relocating [22, 23, 25, 26]. This present research sought to address this gap by providing a broader examination of the topic. Furthermore, the study aimed to investigate which specific types of care should be relocated from GPs to other places, a facet not extensively explored in prior research.

Countries worldwide are encountering similar challenges: escalating demand for healthcare, rising costs, and dwindling health workforce [1–6]. Consequently, the findings of this study are not only pertinent to the Netherlands but also hold relevance for other nations with comparable healthcare systems, particularly those with a strong primary care.

### Strengths and limitations

Notably, GPs aged 60 or older, who were part of the interview process, often expressed distinct viewpoints on relocating care compared to other participants. It is important to acknowledge that GPs aged 39 years or younger were not included in our study cohort, and their perspectives on relocating care could possibly contribute different insights. The older GPs observed that their younger counterparts perceived the pressures within general practice as even more significant due to the balancing act between work and family. This disparity in perception might influence their perspectives on care relocation and could elucidate the absence of GPs under the age of 39 in this study. It is conceivable that heavier workloads experienced by younger GPs may limit their availability or inclination to participate. Furthermore, several older GPs mentioned that they had slightly reduced their working hours, affording them the flexibility to engage in such activities. For future research, it is important to recruit participants through theoretical sampling so that younger GPs, and GPs with less experience, are also represented. Further research could be carried out on the differences between the perspectives of younger and older GPs.

Also the sample of Dutch GPs in the study was a non-random one. Some overrepresentation can be expected of GPs with a higher than average interest in the topic of this study. This is especially true because all the GPs were recruited through Nivel’s network on eHealth and innovation. As a result, there may be a certain degree of overrepresentation of GPs who are enthusiastic about relocating care, who maintain an open-minded perspective about it, or have the availability to participate in such studies and thus generally have more time and are positive about taking on new tasks. However, both GPs who were enthusiastic, as well as GPs who were very critical of relocating care, participated during the interviews. We, therefore, think that potential overrepresentation did not influence the results of the study.

We reached data saturation during the interviews for the GPs aged 40 years and older, which is a strength of this study. During the data coding process, new data was continuously compared with previously collected data to discern similarities and differences. After coding seven interviews, we reached a point where no new codes emerged, indicating that we reached data saturation. Furthermore, the inclusion of the exploratory focus group with six GPs preceding the interviews enhanced the quality of the study. This approach facilitated that the interview guide aligned well with the GPs during the interviews. It had a coherent structure where the GPs could express their views while ensuring that all relevant questions were addressed. Additionally, insights gleaned from the exploratory focus group were corroborated during the subsequent interviews, lending further credibility to our findings. Finally, another strength of the research is that we worked with a research team of four researchers from different backgrounds who collaborated closely allowing triangulation of the data analyses and peer discussions.

### Future research

In this study, GPs identified which types of care should be relocated and outlined conditions necessary to achieve this relocation. Future research could concentrate on a specific category of care relocation, mentioned by the GPs and explore strategies for its implementation. We recommend commencing with a focus on relocating certain responsibilities from GPs to alternative healthcare settings, such as addressing the issue of the unnecessary referrals they are required to write. Subsequent research could delve into the frequency of such occurrences, investigate the underlying causes, and develop effective methods for organizing the relocation of this particular type of care.

## Conclusion

In order to organize the relocation of care from the hospital to the GP, such as follow-up care and diagnostic care, the most important condition is that care should first be relocated from the GP to other settings. Several participants emphasized that, currently, they lack the necessary time to assume tasks from hospitals. Therefore, this preliminary measure entails freeing up capacity by relocating care away from GPs and towards other settings. Care that could be relocated away from the GP is care where the knowledge of the GP adds no value, care that ends up with the GP due to navigational problems, and care that can be managed through self-measurements.

### Electronic supplementary material

Below is the link to the electronic supplementary material.

Supplementary Material 1: Exploratory focus group topic list/interview guide

Supplementary Material 2: Interviews topic list/interview guide

Supplementary Material 3: Codebook

## Data Availability

The qualitative data collected and analysed during the current study concerns individual interview reports with GPs which fall under the GDPR and are therefore not publicly available. However, they are available from the corresponding author on reasonable request.

## References

[1] Organisation for Economic Co-operation (2021). And Development. OECD work on health.

[2] Rudnicka E, Napierała P, Podfigurna A, Męczekalski B, Smolarczyk R, Grymowicz M (2020). The World Health Organization (WHO) approach to healthy ageing. Maturitas.

[3] Rechel B, Doyle Y, Grundy E, McKee M. How can health systems respond to population ageing? Technical report. Copenhagen: World Health Organization; 2009. Report No.: 10.

[4] Liu JX, Goryakin Y, Maeda A, Bruckner T, Scheffler R (2017). Global Health Workforce Labor Market Projections for 2030. Hum Resour Health.

[5] Organisation for Economic Co-operation and Development (2021). Health at a glance 2021: OECD indicators.

[6] Boniol M, Kunjumen T, Nair TS, Siyam A, Campbell J, Diallo K. The global health workforce stock and distribution in 2020 and 2030: a threat to equity and ‘universal’ health coverage? BMJ Glob Health. 2022;7(6). 10.1136/bmjgh-2022-009316 PMC9237893 35760437

[7] Organisation for Economic Co-operation and Development (2017). Tackling wasteful spending on Health.

[8] Barrenho E, Haywood P, Kendir C, Klazinga NS. International comparisons of the quality and outcomes of integrated care: Findings of the OECD pilot on stroke and chronic heart failure. 2022.

[9] Westra D, Kroese M, Ruwaard D (2017). Substitutie Van Zorg. Nederlands Tijdschrift Geneeskunde.

[10] Taskforce De Juiste Zorg Op de Juiste Plek (2018). De Juiste zorg op de juiste plek rapport: Wie durft?.

[11] Heida JP, Hoendervanger J (2016). Next level gezondheidszorg: hoe de zorg efficiënter en beter Kan.

[12] Strategies in Regulated Markets (SiRM) (2021). Substantieel potentieel. Schatting Van De potentiële Opbrengst Van Substitutie Van zorg en inventarisatie Van de benodigde voorwaarden.

[13] Beech J, Gardner G, Buzelli L, Williamson S, Alderwick H. Stressed and overworked. Health Foundation; 2023.

[14] World Health Organization. Evidence, policy, impact: WHO guide for evidence-informed decision-making. 2021.

[15] van Hoof SJM, Quanjel TCC, Kroese M, Spreeuwenberg MD, Ruwaard D (2019). Substitution of outpatient hospital care with specialist care in the primary care setting: a systematic review on quality of care, health and costs. PLoS ONE.

[16] Cryer L, Shannon SB, Van Amsterdam M, Leff B (2012). Costs for ‘hospital at home’ patients were 19% lower, with equal or better outcomes compared to similar inpatients. Health Aff (Millwood).

[17] Gupta Strategists (2016). No place like home. An analysis of the growing movement away from hospitals towards providing medical care to patients in their own homes.

[18] Damen LJ, Van Tuyl LHD, Korevaar JC, Knottnerus BJ, De Jong JD (2024). Citizens’ perspectives on relocating care: a scoping review. BMC Health Serv Res.

[19] Meiklejohn JA, Mimery A, Martin JH, Bailie R, Garvey G, Walpole ET (2016). The role of the GP in follow-up cancer care: a systematic literature review. J Cancer Surviv.

[20] Easley J, Miedema B, Carroll JC, Manca DP, O’Brien MA, Webster F (2016). Coordination of cancer care between family physicians and cancer specialists: importance of communication. Can Fam Physician.

[21] Schütze H, Chin M, Weller D, Harris MF (2018). Patient, general practitioner and oncologist views regarding long-term cancer shared care. Fam Pract.

[22] Van Hoof SJ, Spreeuwenberg MD, Kroese ME, Steevens J, Meerlo RJ, Hanraets MM (2016). Substitution of outpatient care with primary care: a feasibility study on the experiences among general practitioners, medical specialists and patients. BMC Fam Pract.

[23] Van Hoof SJM, Kroese MEAL, Spreeuwenberg MD, Elissen AMJ, Meerlo RJ, Hanraets MMH et al. Substitution of hospital care with primary care: defining the conditions of primary Care Plus. Int J Integr Care. 2016;16(1). 10.5334/ijic.2446 PMC5015530 27616956

[24] Albada T, Berger MY, Brunninkhuis W, van Kalken D, Vermeulen KM, Damstra RJ (2023). A care substitution service in the Netherlands: impact on referral, cost, and patient satisfaction. BMC Prim Care.

[25] Noels EC, Wakkee M, van den Bos RR, Bindels PJE, Nijsten T, Lugtenberg M (2019). Substitution of low-risk skin cancer hospital care towards primary care: a qualitative study on views of general practitioners and dermatologists. PLoS ONE.

[26] Firet L, de Bree C, Verhoeks CM, Teunissen DA, Lagro-Janssen AL (2019). Mixed feelings: general practitioners’ attitudes towards eHealth for stress urinary incontinence-a qualitative study. BMC Fam Pract.

[27] Liemburg GB, Korevaar JC, van Zomeren WT, Berendsen AJ, Brandenbarg D (2022). Follow-up of curatively treated cancer in primary care: a qualitative study of the views of Dutch GPs. Br J Gen Pract.

[28] Kringos D, Boerma W, Bourgueil Y, Cartier T, Dedeu T, Hasvold T (2013). The strength of primary care in Europe: an international comparative study. Br J Gen Pract.

[29] Guest G, Bunce A, Johnson L (2006). How many interviews are enough? An experiment with data saturation and variability. Field Methods.

[30] Braun V, Clarke V (2006). Using thematic analysis in psychology. Qualitative Res Psychol.

[31] Nowell LS, Norris JM, White DE, Moules NJ (2017). Thematic analysis: striving to meet the trustworthiness criteria. Int J Qualitative Methods.

[32] Tong A, Sainsbury P, Craig J (2007). Consolidated criteria for reporting qualitative research (COREQ): a 32-item checklist for interviews and focus groups. Int J Qual Health Care.

[33] CCMO. Your research: Is it subject to the WMO or not? 2022 [cited 2022 11 July 2022]. https://english.ccmo.nl/investigators/legal-framework-for-medical-scientific-research/your-research-is-it-subject-to-the-wmo-or-not.

[34] CBS. Stedelijkheid (van een gebied): CBS. 2024 [cited 2023 29-08-2023]. https://www.cbs.nl/nl-nl/onze-diensten/methoden/begrippen/stedelijkheid--van-een-gebied.

[35] Dutch College of General Practitioners. GPinfo.nl: Dutch College of General Practitioners. 2023 [updated 2023; cited 2023 25 August 2023]. https://gpinfo.nl/.

[36] Augestad KM, Norum J, Dehof S, Aspevik R, Ringberg U, Nestvold T et al. Cost-effectiveness and quality of life in surgeon versus general practitioner-organised colon cancer surveillance: a randomised controlled trial. BMJ Open. 2013;3(4). 10.1136/bmjopen-2012-002391 PMC3641467 23564936

[37] Mittmann N, Beglaryan H, Liu N, Seung SJ, Rahman F, Gilbert J et al. Examination of Health System resources and costs Associated with transitioning Cancer survivors to Primary Care: a propensity-score-matched cohort study. J Oncol Pract. 2018:Jop1800275. 10.1200/JOP.18.00275 30289736

[38] Baena-Cañada JM, Ramirez-Daffos P, Cortes-Carmona C, Rosado-Varela P, Nieto-Vera J, Benitez-Rodriguez E (2013). Follow-up of long-term survivors of breast cancer in primary care versus specialist attention. Fam Pract.

[39] Doty MM, Tikkanen R, Shah A, Schneider EC (2020). Primary Care Physicians’ Role in Coordinating Medical and Health-Related Social needs in Eleven Countries. Health Aff (Millwood).

[40] Van Dulmen SA, Kruse FM, Burgers JS. Primary health care through the eyes of the general practitioner; an international study. Ned Tijdschr Geneeskd. 2021;165. 33914426

[41] Van der Horst HE, De Wit N (2020). Redefining the core values and tasks of GPs in the Netherlands (Woudschoten 2019). Br J Gen Pract.

