# Supplementary material for: General practitioners’ perspectives on relocating care: a Dutch interview study
Source: BMC Prim Care. 2024 May 25;25:186. doi: 10.1186/s12875-024-02425-1 (PMC11127345; doi:10.1186/s12875-024-02425-1)
Supplement: Supplementary file 1 — Supplementary Material 1: Exploratory focus group topic list/interview guide [file 12875_2024_2425_MOESM1_ESM.docx]

**Appendix A – Exploratory focus group: topic list/focus group guide**

1. Could you introduce yourself?
2. What motivated you to participate in this focus group?
3. What does "the right care in the right place" mean to you? How do you define the ideal location for receiving care?
4. Which care can be relocated and why?

- What are the defining characteristics of care that can be relocated?
- Are there any types of care that you believe cannot be relocated? If so, what are they?
- Can you provide examples of specific diagnoses where relocating care might be feasible?
- When considering relocating care, which specific groups come to mind?

1. Under which conditions can care be relocated?
   *Skills, knowledge, non-life-threatening situation, role of patients*

- What resources do you believe are necessary to facilitate the relocation of care?
  *Financial resources, personnel, equipment, time.*
- Which HCPs should play a role when relocating care?
- Apart from healthcare professionals, which other parties or stakeholders do you think should be involved in the process of relocating care?
- What do you perceive as the main obstacles or challenges when it comes to relocating care?
  *Lack of resources, skills knowledge, patients trust, disengagement of certain HCPs/parties*
