# Supplementary material for: General practitioners’ perspectives on relocating care: a Dutch interview study
Source: BMC Prim Care. 2024 May 25;25:186. doi: 10.1186/s12875-024-02425-1 (PMC11127345; doi:10.1186/s12875-024-02425-1)
Supplement: Supplementary file 2 — Supplementary Material 2: Interviews topic list/interview guide [file 12875_2024_2425_MOESM2_ESM.docx]

**Appendix B – Interviews topic list/interview guide**

1. Could you introduce yourself and provide some information about the practice where you work?
   *Age, experience, how urban is the practice setting, kind of practice*
2. Why did you choose to participate in this interview?
3. Which care can be relocated from the GP to other settings?

- Which care can be relocated to self-care?
  *Examples, which kind of complaints could be relocated, conditions, who should be involved*
- Which care can be relocated to eHealth?
  *Which form of* eHealth*, examples, which questions could be relocated, conditions, does it relieve the workload*
- Which care should be relocated to other healthcare providers?
  *Why is this care not already relocated? What goes wrong in this moment? Conditions; Who should be responsible?*

1. Which care can be relocated from the hospital to the GP?
   *Which diagnoses, which treatments, examples, skills needed, conditions*

- What care should be relocated from the patient’s point of view?
- What do you think about one-and-a-half-line care?
  *Do you use it? Can it be used more? And on which treatments or conditions?*
