# Supplementary material for: General practitioners’ perspectives on relocating care: a Dutch interview study
Source: BMC Prim Care. 2024 May 25;25:186. doi: 10.1186/s12875-024-02425-1 (PMC11127345; doi:10.1186/s12875-024-02425-1)
Supplement: Supplementary file 3 — Supplementary Material 3: Codebook [file 12875_2024_2425_MOESM3_ESM.docx]

**Appendix C – Codebook**

- Care that can be relocated from the GP to other places
- Why?
- Conditions
- Examples
- Care that can be relocated from the hospital to the GP
- Why?
- Conditions
- Examples
- Care that is already relocated
- Target group suitable for relocation
- Cause more/less pressure GP
- Other
